# Observations on the History and Treatment of Dysentery and Its Combinations

**Published:** 1847-07-01

**Authors:** 


Observations on the History and Treatment of Dysentery and its Combinations; with an Examination of their Claims to a Contagious Character, and an Enquiry into the Source of Contagion in its analogous Diseases, Angina, Erysipelas, Hospital Gangrene, and Puerperal Fever. By *William Harty*, M.D., &c. 2nd Edition, 8vo. pp. 303. Hodges and Smith, Dublin, 1847.

The first edition of this truly valuable work was published more than forty years ago, and received a most favourable character from the leading medical Journals of that time: it was mentioned also with commendation by Sprengel in his Critical Review of Medicine. To most of the readers of the present day it has all the novelty of a new book; few are acquainted with it, and fewer still have learned to appreciate the value of the doctrines it propounds. To us it has a peculiar interest; and more especially at the present time, when we have been endeavouring to settle in some degree the opinions of our medical brethren on the important subject of Infection, as an attribute of certain diseases, and a cause of their propagation and diffusion. It is, indeed, most gratifying to find that so enlightened an observer as the author of the work now under review had long ago adopted and advocated, with no common ability, the same view of the question as we have sought to establish: the sanction of such an authority goes far to confirm us in the accuracy of our own opinions. In alluding to the subject of Contingent Infection, in the last number of this Journal (p. 459), we gave expression to the following remark, in reference to the very disease upon whose true history Dr. Harty has happily thrown so much light:—"If the broad and general question, Is Erysipelas or is Dysentery infectious? were put to a physician, he might with perfect truth and propriety answer it in the affirmative; and yet all that he meant by such a response might be the simple declaration of his opinion that these diseases are occasionally or conditionally communicable from one person to another, although such an occurrence does not happen once in a thousand times, and only under very peculiar circumstances." In the present article we shall have an opportunity of explaining what is the nature of those circumstances, to which allusion was then made.

In commencing our observations, we may remark that there is perhaps no malady on which there has been greater discordancy and contrariety of opinion as to many of its most important phenomena and attributes than Dysentery; and, judging from the observations of the very latest writers upon this disease, medical men seem to be nearly as far remote from settled or well-grounded opinions on some points, as ever. Dr. Copland has said—with too much truth we fear—that, "our knowledge of the disease is but little in advance of what it was two centuries ago, and that even the most recent writers are distinguished rather by confined and exclusive ideas as to its nature and treatment than by comprehensive views of its forms and manifestations in connexion with the various combinations of causes producing it, and the diversified circumstances in which it prevails." And then he adds the philosophic remark:—"Exclusive notions of a disease are the result of a knowledge merely of what has occurred within the sphere of the author's observation; whilst more extended ideas are acquired from what he has remarked in various climates, on different occasions, and at distant periods, and from an acquaintance with what has been observed by others, believing, truly, that nothing is constant but change; that what has occurred or prevailed formerly will recur again; and that one form is as likely as another to appear in future, whenever the concurrence of causes, of which it is a necessary or contingent result, shall take place.".......... "That writer but imperfectly performs his duty, who, in giving a history of a most dangerous malady, confines himself to the particular form it has assumed during a few seasons, within the single locality of which he is the centre, and argues that it is always as he observed it: thereby affirming as true of the genus, what may be hardly true of the species."* So much for one high authority; let us see what says another.

In the last edition of the classical work of Dr. Johnson and Mr. Martin on the "Influence of Tropical Climates, &c." we read:—"There is hardly a disease in the whole range of nosology, regarding which so great a discrepancy among authors and practitioners has existed as in dysentery; and this must have originated, I conceive (says Dr. J.) in consequence of mistaking prominent *effects* for proximate *causes;* and, as the means of cure directed against the former have often removed the latter, each individual believed that he alone had found out the true cause and cure of the disease."

---

* *Dictionary of Practical Medicine,* Vol. I., p. 701.

But it is not so much in reference to the pathology and treatment of dysentery, as to its occasional attribute of infectiousness, that we have now to invite the attention of our readers. As upon this point, too, the utmost confusion still prevails, we sincerely trust that the present analysis of the instructive volume before us will be found to disentangle many of its perplexities.

Dr. Johnson has, in the work just cited, given the weight of his authority in too unqualified a manner to the negative side of the question, resting his opinion solely and exclusively on the results of his own experience in a tropical country; where the disease, it is admitted, seldom exhibits an infectious character.* The most recent writer on Dysentery, Dr. Parkes (whose work† we noticed in our Number for October last), is more guarded in his opinion, and thereby more correct.

"Contagion," says he, "undoubtedly does not act in common dysentery; but it is certain, if the statements of many esteemed authors are to be admitted as evidence, that, at times, in slave ships, and after some campaigns, or in beseiged cities, asthenic dysentery has become contagious, being then complicated with a low fever. This low or typhoid fever has been supposed by some to be the cause of the contagion; but to this is replied, why should the dysentery be always the resulting disease, and not the fever? But it is a question if contagious dysentery is ever seen separated from the accompanying fever; are not the two diseases always propagated together? Are there instances known in which pure uncomplicated dysentery, sthenic or asthenic, as the case may be, has been derived from a case compounded of dysentery and typhus?" P. 132.

Dr. Parkes is quite right in his conjecture that it is the association or conjunction of Typhus fever with Dysentery that renders the latter disease liable to become infectious. This point in its history Dr. Harty has elucidated far more ably and successfully than any writer, either before or since the publication of the first edition of his work. As we have said, his views have not hitherto been appreciated by the profession as they deserve. Had they been better known and understood, there would doubtless have been less wavering and discrepancy in the statements of many writers—and those, too, men of experience—during the course of the present century.

The following three positions contain the pith and marrow of our author's opinions on the general history of the disease we are now considering:—

1st.—*That the genuine and simple dysentery is unattended by idiopathic fever, and is never of itself contagious (infectious).*

2nd.—*That every form of the disease, when epidemic, is a combination of the simple dysentery either with intermittent, remittent, or continued fever; and,*

---

* It would seem, however, from the following extract from the Medico-Chirurgical Review for October 1838, quoted by Dr. Harty, that Dr. Johnson did admit the occasional or contingent infectiousness of Dysentery. "We doubt much whether the disease is ever infectious *per se*, but only when it is an attendant on a low or typhoid fever, when the latter may prove infectious, and carry its intestinal character from person to person."

† *Remarks on the Dysentery and Hepatitis of India.* 1846.

3rd.—*That the combination with continued fever alone is contagious (infectious).*

Having illustrated in, as appears to us, the most satisfactory manner, the truth of these conclusions, Dr. H. then proceeds to examine the subject of the Treatment of the different forms of Dysentery, pointing out as he goes along how necessary it is to have clear and accurate notions on the above pathological positions, if we hope to attain to anything like precision or accuracy in our therapeutic principles. Thus, the practical importance of the preliminary enquiry is rendered obvious, while, at the same time, fresh proofs of the accuracy of the conclusions arrived at are obtained from an examination of the remedies that have been found to be most useful at different times.

The first Chapter is devoted to the consideration of *Simple Dysentery.* By this term our author wishes to be understood that species of the disease in which the intestinal symptoms—viz. severe tormina of the bowels, frequent dejections of a mucous and bloody character, accompanied with a most distressing tenesmus—are not necessarily associated with any form of *idiopathic* fever. That fever is not an essential accompaniment of Dysentery, is abundantly obvious from the observations of some of the most accurate describers of the disease. Sydenham states that, in the majority of cases, which came under his notice, the gripes attack first, and the purgings succeed, without being preceded by any fever. Akenside* asserts that dysentery is very rarely attended by fever, not once in ten cases of the disease. Stoll† too, when speaking of the rheumatic-bilious form of the disease, says, " febre quidem evidenti carebat, non omni tamen motu febriculoso." Roederer‡ says, " fever was either altogether absent, or it set in only after the disease had existed for some time : few patients sickened from the first with febrile symptoms." M. Vignes|| also, when describing the dysentery prevalent in the French army encamped around Vienna in 1809, states that, in the Spring, " in several cases we noticed the prevalence of slight fever, but in the majority there was none at all. In the latter the symptoms were always less severe, than in the former." As the season advanced and numbers increased, " the dysentery began to be complicated with typhus, adynamia, ataxia, or nervous fever, which prevailed epidemically throughout the army ; it spread also amongst the inhabitants of the country. Among the latter, however—and this is a fact especially well worthy of notice—the disease appeared only under the characters of the fever, the dysenteric symptoms not occurring in them." From this circumstance, M. Vignes infers that the intestinal affection was generally owing to the exposure to inclement and variable weather, excessive fatigue, and the insufficient or irregular supply of food ; from all which evils, the soldiers in the French army suffered more severely than the people of the country. Frank§ also, who had very ample experience

---

* *De Dysenteriâ Commentarius.* Lond. 1764.
† *Ratio Medendi.* Vien. 1788.
‡ *De Morbo Mucoso.* Gotting. 1783.
|| *Traité Complet de la Dysenterie.* Paris, 1825.
§ *Epitome de Curandis Hominum Morbis.* Lib. v. *de Profluviis.* Vien. 1807.

in the disease, states that "in numerous cases there is no shivering nor feverish heat at its commencement, no loss of appetite is experienced, and there is scarcely any perceptible acceleration of the pulse." Dr. Parkes, too, says :—" In some particular forms there may be attendant feverishness ; but in the majority of cases there is no pyrexia, in the common acceptation of the term. Often there are no rigors or pains in the limbs, or any affection of the general health."

The passages we have now quoted—and their number might easily be multiplied threefold—will sufficiently serve to shew that fever is not a necessary or essential accompaniment of an attack of dysentery. That a greater or less degree of febrile excitement very generally supervenes, if the symptoms be not immediately arrested, will be denied by no one; but this is very different from fever being supposed to constitute one of the primary characters of the disease. Hence its definition, given by Cullen, is clearly erroneous. It stands thus :—" *Pyrexia contagiosa; dejectiones frequentes, mucosæ, vel sanguinolentæ, retentis plerumque fæcibus alvinis; tormina, tenesmus.*" We shall not now allude to the (alleged) fever being here declared to be always infectious ; as this subject will engage our attention in the sequel. Akenside's descriptive definition is altogether more accurate :—" *Qui graviora patitur ventris tormina, simul cum frequente desidendi cupiditate, et cum dejectionibus vel sanguineis vel mucosis (vel utrisque), eum hominem Dysenteria laborare omnes consentiunt medici.*"

Simple Dysentery is very generally the result of exposure to wet and cold in the months of Summer or Autumn, more especially when this most prolific cause of disease is aided by the use of unwholesome or insufficient food, acid unripe fruits, pernicious drinks, and, in short, of whatever has a tendency to induce irritation of the mucous surface of the bowels. Almost all sporadic cases of the malady belong to the *simple* form now described; and instances of it are also far from uncommon in every epidemic, whatever be the prevailing type of the fever by which the epidemic may be characterised. Before proceeding to notice those forms of the disease which arise from a combination of the intestinal affection with fever of different types, Dr. Harty devotes a chapter to illustrate the *Affinity between Dysentery and Rheumatism.*

"The opinion that there exists an intimate connexion between these diseases, first suggested by Alexander of Tralles (who called dysentery a rheumatism of the intestines), has in modern times been adopted by Akenside, Richter, Stoll, and others. Independently of the authority of these writers in favour of this doctrine, we find in other authors a few scattered facts, which must carry the greater weight, as they are recorded by individuals unbiassed by any theory on the subject." P. 22.

Akenside states that he often observed rheumatism to supervene upon the decline of dysentery and *vice versâ*, that occasionally the two diseases exist together, and that both disappear under the same line of treatment. Every physician must have remarked that the stools in the former disease often exhibit the dysenteric character, consisting almost entirely of mucus, and attended with griping pains and distressing tenesmus.*

---

* Daily experience shews that not only rheumatic, but gouty disease also, is

Dr. A. mentions several cases of the apparent conversion of the one disease into the other, or at least of their alternating with each other. In one of these cases, the patient had three returns of rheumatism, and as many of dysentery, before he was restored to health. After adducing a variety of details, he concludes thus:—" I have so often had occasion to observe the resemblance between the two diseases, that now I regard Dysentery as a Rheumatism of the bowels, and fairly believe that the cause of both is alike." Stoll held the same opinion and uses nearly the same words to express it. He calls rheumatism and dysentery *παθηματα αδελφεα*, and assigns these reasons for this belief:—1. Because he often observed dysentery to supervene upon the sudden cessation of rheumatism; 2. Because sometimes the one and sometimes the other disease occurred in the same individual; and 3. Because dysentery often suddenly ceased as soon as any of the joints became swollen and painful." Richter† too declares himself satisfied of the truth of these positions, and has related several instances of the mutual conversion of the two diseases. Zimmermann‡ also may be quoted to the same effect; he mentions several cases where rheumatic symptoms immediately supervened upon a sudden arrest of dysenteric symptoms from the use of opiates and other astringents. Tissot|| and Sir George Baker§ make a similar remark. Hoffman had at an earlier period observed that, when dysentery is quickly arrested by the use of astringents and opiates, among other evil consequences, severe articular pains are apt to be induced. M. Vignes mentions that, among the numerous cases occurring in the French army around Vienna, " the alvine dejections and other dominant symptoms of the dysentery sometimes suddenly ceased upon the supervention of rheumatic pains in the knees, elbows, wrists and shoulders." Frank says nearly the same thing: " in some patients the pain of the joints seems to have freed the bowels from suffering."

It will have been perceived, from the preceding statements, that the supervention of rheumatic disease in cases of dysentery has generally taken place, when the intestinal discharges have been suddenly or prematurely checked. We may hence derive a most useful therapeutic precept, viz. not to have recourse to astringent medicines at once and without any preliminary treatment.

It may be here worthy of notice, that Frank has drawn an ingenious analogy between Dysentery and Cynanche. Thus: " It is the same sort

---

occasionally apt to induce intestinal fluxes as well as catarrhal affections. The cause would seem to be that there is present, in these morbid states, an acrimonious peccant material in the blood, which requires to be eliminated and discharged from the system; and if this be not readily carried off by the appointed emunctories—viz. the kidneys and skin—it is liable to fall upon the mucous lining of the alimentary canal or of the air-passages. Hence it is that the above diseases are not unfrequent concomitants of Oliguria or deficiency of urine, from whatever cause this originates.—*Rev.*

† Observations, Medical and Surgical. 1794.

‡ *On Dysentery.* Translated by Dr. Hopson from the German. Lond. 1771.

|| *Avis au Peuple.* Translated by Kirkpatrick. 1771.

§ *De Catarrho et de Dysenteriâ Londinensi.* 1764.

of disease which attacks the throat or upper end of the alimentary canal, and the anus or its lower end: there is the strongest affinity between Dysentery and many species of Cynanche. In severe cases of the latter, we have the sense of burning, the redness, swelling, tension, the discharge of a puriform matter that is sometimes tinged with blood, the constant and painful effort of swallowing, not unlike to the tenesmus that occurs in the former." In a subsequent passage, he points out the striking similitude between the malignant forms of the two diseases.*

M. Bruant, in his official report on the dysentery prevalent in the French army in Egypt, takes notice of a curious sympathy between the pains of chronic Dysentery and those of Ophthalmia: "The affection of the eyes always brought a marked relief, when it occurred in the course of long-standing dysentery; the pains of the eyes and those of the abdomen mutually took the place one of the other; the latter, however, usually re-appeared after the cessation of the former."

We now pass on to the consideration of a very important point in the history of the disease.

### Chap. III.—*Combinations of Dysentery; and first of the Intermittent and Remittent Forms.*

Morton,† in his excellent account of the epidemic dysentery which proved so destructive in London about the middle of the 17th century, expressly says that the bowel affection was only a symptom of the remittent fever, which was in fact the primary disease. So convinced was he, from the distinct remissions and exacerbations observable every or every second day, of the truth of this position, that he proposed that the dysentery should be designated by the appellation of spurious or colliquative Συνεχης.‡

Sydenham, it is well known, has, in more passages than one, spoken of dysentery as a "febris intraversa," or fever turned in upon the bowels. There has been a good deal of difference of opinion as to the exact meaning of this expression. But, whatever this may be, it very significantly

---

* Sydenham, in his description of dysenteric fever, has alluded to the alliance which he observed, one year, between the intestinal affection and rheumatism and cynanche conjointly: "The fever of 1671 was constantly attended, towards its decline, with extreme sickness, a vomiting of green bile, and a marked tendency to diarrhœa; while that of the following year was accompanied with pains in the muscular parts of the body, especially in the limbs, resembling those of *rheumatism,* and also with *inflammation of the throat,* but of a milder character than is present in regular quinsy. Yet both these met in the same specific fever, and both required the same method of treatment; they differed only by respect, or in consequence, of the sensible qualities of the air that prevailed at the time when these phenomena arose."—*Observ. Med.* IV. 4, 6.

† De Febribus. Lond. 1693.

‡ Cullen, in his synopsis, includes the Febris Syneches Epidemica ab anno 1658 ad 1664, et postea ab anno 1673 ad 1691, described by Morton, under the head of tertian remittents. The reader should be on his guard not to mistake the fever termed Συνεχης, for that which is so well known by the name of Συνεχος, or common continued fever.

implies the intimate connexion that this truly Hippocratic observer found often to subsist between the two diseases in question, whether this connexion is evidenced by the one becoming convertible into, or taking the place of, the other, or in any other manner. And as we know that the fever, to which Sydenham alludes under that term, was of the remittent type, the evidence of our great countryman may be fairly quoted here in proof of the subject matter we have in hand.

It may be worthy of notice, *en passant*, that Sydenham mentions that in the first Autumn, when the dysentery he saw prevailed, many patients had no purging whatever; but that, in respect of the severity of the gripings, the intensity of the fever, the sudden prostration of the strength, and other symptoms, the disease vastly surpassed the dysenteries of subsequent years.

Willis,* too, has left a very accurate description of the dysentery which proved so wide-spread and destructive in London during the years 1670, 1671. The cholera had raged, it may be observed, with great severity in the early part of the Autumn of the latter year. About the Equinox there arose an epidemic intermittent or remittent fever, which had in some cases a quotidian, in others a tertian, type. " While this fever prevailed in the villages and towns throughout the country, the bloody flux committed great ravages in London." Willis attributes many of the characteristic features of this flux to the epidemic fever spread over the land, just as Sydenham did to his dysenteric fever; so that these two fevers would appear to be identically the same.

Moseley† testifies to the general truth of Sydenham's opinions, remarking that, " as the flux conforms by the number of stools, and by its rapidity, to the degree, so it does to the state of the fever of the season, when it prevails; the stools being more frequent, and all the symptoms becoming more aggravated at those hours when the current fevers are in their exacerbation, and the reverse when these fevers are in their remissions." Now we need scarcely say that it is this very character of having distinct remissions and exacerbations every, or every other, day, that has led so many of the most experienced and enlightened observers to regard the dysentery, on various occasions, rather as one of the symptoms of a periodic fever than as a primary and essential disease.

Cleghorn‡ observed so marked a resemblance between dysentery and intermittent fever, that he was led to exhibit bark for the cure of the former. " When the fever and gripes," he says, " were regularly exasperated every day, or every other day, at stated periods, the bark has often effectually put a stop to both, especially if the exacerbation began with chilliness, and terminated in sweats: *at other times it removed the fever, the flux continuing without much alteration.*"

Roederer, too, when speaking of the relation between the two diseases, mentions a striking instance of their combination:—" During this year, many patients were affected with intermittent fever and with dysentery

---

* Opera Medica. Lugdun. 1676.

† On Tropical Diseases. Lond. 1803.

‡ On the Diseases of Minorca. 1762.

at the same time, or with what might be called a genuine dysenteric ague. In a neighbouring town, however, the fever only prevailed epidemically; in another, which was more remote and surrounded by hills, the dysentery alone existed; while in a third and intermediate one there were but very few cases of either disease."*

Clark† also, in his description of the dysentery, or, as he calls it, the dysenteric fever of Bengal, has remarked that the intestinal affection seemed rather to be a symptom of the endemic remittent fever of the country, than an original disease. Hunter,‡ too, says that between dysentery and the remittent fever in Jamaica, there subsists an intimate connexion, the one frequently changing into the other, and both being often complicated with various degrees of violence. Nicholl states that, at Seringapatam, the disease was frequently combined with remittent and intermittent fevers; and Trotter§ has remarked the same thing with respect to the coast of Africa and the West Indies. Even in our own climate, Willan|| has mentioned that, in the worst cases of the epidemic of 1800, (a year remarkable for cold, wet, and famine,) a considerable degree of fever prevailed from eight to ten days; the pulse 100 in the morning, and 120 in the evening; there was constant flushing of the face and coldness of the extremities; and a periodical aggravation of pain for three or four hours every forenoon was to be observed.

Frank also has alluded to the frequent connexion of the two diseases. "I have seen," says he, "dysentery follow upon periodic fever, and put a stop to it; and no sooner was the dysentery cured, than the intermittent returned as before. Indeed, the former seemed but as a symptom of the latter, and speedily vanished when the producing cause was got rid of." Dr. Wilson, in his recent interesting volume on China, says:—"In truth, the flux ought to be considered a constituent part of the periodic fever, rather than an independent malady in most instances."

The evidence now adduced is surely sufficient to prove, beyond all dispute, the not unfrequent connexion of Dysentery with Periodic Fevers;—

---

* Degner* has remarked that the fever, which accompanied the dysentery which he saw, sometimes exhibited the character of an irregular tertian; and "this," he adds, "appears to me the reason why, in certain dysenteric patients, the symptoms were always worse on every third day." Huxham,† in his account of the epidemic dysentery prevalent in England in 1743, says:—"The disease was generally associated with fever, or perhaps I should rather say, it was a symptom of this fever; from its very commencement, and before the supervention of any tormina in the bowels, there was usually no inconsiderable feverish heat, with quickened pulse, and dryness of the tongue. Most frequently, it attacked with a distinct shivering, and, in many instances, it exhibited the characters of a tertian fever."

* *De Dysenteriâ Bilioso-contagiosâ. Trajecti ad Rhenum.* 1754.

† *De Aere et Morbis Epidemicis.* 1752.

† *On Diseases of Hot Climates.* 3d ed. 1809.

‡ *On the Diseases of the Army in Jamaica.* 1778.

§ *Medicina Nautica.* Lond. 1799—1803.

|| *Reports on the Diseases in London.* 1801.

a point, be it remembered, of the highest importance, as respects the treatment of the disease. That there are some anomalies in the nature of this connexion must be admitted; they are alluded to in the following statement of our author:—

" Dr. Buel describes a bilious fever and dysentery as prevalent in Sheffield (State of Massachusetts), in 1796, arising from the obvious influence of marsh miasmata, and states that in some 'the dysentery came on while the patient was affected with the fever, in which case the type of the fever soon became obliterated, and the accompanying febrile symptoms were similar to those in original dysentery.' 'Sometimes the fever came on upon the dysentery: the type of the fever was not in this case easily ascertained, until an abatement of the dysentery took place, when, as the dysenteric symptoms subsided, the fever would appear in its proper form. The two disorders appeared to be complicated; that is, they both seemed to exist at the same time, rather than to act in alternation.' 'In this sickness,' he adds, 'there is every reason to ascribe identity of cause to the two disorders: they were circumscribed in a very striking manner by precisely the same limits, and they both began and ceased to prevail at the same time.' Dr. B. was convinced that 'neither of these diseases was propagated by specific contagion.' Dr. Ffirth also, in his Dissertation on Malignant Fever, says that the crew of the ship in which he sailed to Batavia (seventy-six in number), had, with the exception of eight, either marsh fever or dysentery; that the fever appeared to alternate with dysentery; when the weather was bad the latter prevailed; when good, the former.' From this latter statement it is not to be inferred that the two diseases 'alternated' in the same individual, in the same sense that Dr. Buel speaks of their alternation: in this case, when one disease was prevalent, the other was not. Hargrave, too, in his History of the Walcheren Fever, states that 'in proportion as the autumn grew cool, these fevers (bilious remittent) abated of their ardour, and formed more easily into intermittents, though still irregular and of a bad kind. The dysentery was never general, though not uncommon, and it was observable that those who were seized with it usually escaped the fever, or if any had both, it was alternately; so that when the flux appeared the fever ceased, and when the first was stopped, the other returned: whence it appeared that, though the two distempers were of a different form, they proceeded from a like cause.' "* P. 54.

As exposure to cold and wet, in certain climates and under certain unsalutary circumstances, is the common cause of simple dysentery, so the complication of the disease with periodic fever may very fairly be presumed to be owing to the operation of these causes in conjunction with the agency of malaria, the prolific source of remittents and intermittents. How often, nay, how generally, are troops exposed to both of these morbific influences at one and the same time! Can we therefore be " surprised that the causes of both diseases should operate on the same individual, producing a dysentery with distinct intermissions or remissions, when we see that such a combination is frequent in those countries where the diseases themselves often exist independently of each other? This subject is explained after a similar manner by Rollo, who remarks, that the dysentery is produced in place of, or at the same time with, these fevers, when the effluvium or

* " The changes from intermittent fever to dysentery were very common (in the Peninsula), and seemed to suspend the intermittent for a time; but, on the removal of the dysenteric affection, the intermittent returned; in some instances, both diseases attacked the same patient at the same time, and then the dysenteric symptoms were aggravated."—*Sir James MacGregor in Med. Chir. Transactions.* Vol. VI.

marsh miasma is joined in its action by the exterior application of cold and moisture, to which those affected with dysentery have been always previously exposed."

Without positively asserting that marsh effluvia alone are not competent to induce dysentery, Dr. Harty very reasonably argues that "the influence of wet and cold in its production is more clearly, satisfactorily, and universally demonstrable than that of marsh effluvia, and that it is consequently more legitimate to conclude that, as dysentery can occur in every possible relation to remittent fever, either as absent or present with it, as preceding it or consequent upon it, or as remaining after the fever has ceased, or *vice versâ*, so it cannot be likely that both are always produced by one and the same cause, more especially as those exposed to marsh effluvia are at the same time generally exposed to wet and cold."

As long as the fever, which happens to be associated with Dysentery, is of a purely remittent or intermittent type, our author maintains, and with much show of truth, that the disease is not propagable from one individual to another. Most of the authors already quoted, more especially those whose experience has been chiefly in tropical countries, have unhesitatingly adopted this opinion; and some of them, confining their thoughts entirely to what they have witnessed themselves, have gone so far as to deny that dysentery ever exhibits an infectious quality. That facts, however, do not warrant so sweeping and indiscriminate an assertion must be admitted by every one, we should think, who candidly examines the evidence adduced by our author in the fourth, and second section of the fifth, chapter of his work. The former is devoted to the consideration of the *Combination of Dysentery with Typhus.* We shall now give a summary of its leading contents.

Etmuller* alludes in a very expressive manner to the distinction of Dysentery into two forms or species, the mild and the malignant. "The former," says he, "is usually unaccompanied with fever, is not infectious, and occurs only sporadically; whereas the latter is very generally associated with a malignant, and sometimes a petechial and pestilential, fever, prevailing as an epidemic over a larger district, and multiplying itself by a virulent infection."† He adds: "The tongue is whitish, and covered

---

* *Opera Omnia.* Gen. 1697.

† The reader might infer, from such a passage as the above, that whenever dysentery prevails in an *epidemic* form, it is believed to be infectious. But this is not necessarily so; the disease when associated with remittent fever may prevail epidemically, but is not infectious, as long as the fever retains the genuine periodic type. We are the more anxious that the reader attend to this point, as much of the controversy and dispute respecting the infectiousness of epidemic dysentery has arisen from a neglect of it. Such a passage as the following, taken from the extremely valuable Report of the French Academy on the Plague, might easily lead into the error in question. Alluding to the very important difference between Sporadic and Epidemic Plague, the one being admitted by almost every medical man in Egypt to be not communicable from one person to another, whereas there is difference of opinion as respects the other, the intelligent Reporter, M. Prus, goes on to say:—"There is nothing in this opinion, so generally recognised, not only at Alexandria and Cairo, but also at Smyrna and Constantinople, to surprise the medical man, who knows that *sporadic* dysentery is not communicable, while *epidemic* dysentery is often so in a high degree."

with a thick slime; or, if the fever be high, it is parched and black: there is always an extreme prostration of strength. The disease spreads like the plague, is infectious, and is then propagated under the form of a malignant fever." Zimmermann states that "the circumstance, which impresses on a dysenteric complaint the peculiar mark of malignity," is this, that, "with the causes common to the disorder at all times, others are joined which corrupt the humours, very quickly. The malignant dysentery, therefore, is that in which, either from external causes or from a putrid fomes within the body, a malignant fever supervenes; the pathognomonic signs of this species being formed by the symptoms of a malignant fever mixed with the usual symptoms of a dysentery." "It also," says he in subsequent passage, "very often terminates in, or is a concomitant symptom of, putrid and malignant fevers; but, when a malignant fever supervenes on a dysentery before subsisting in the body, this is quite a different case, and constitutes a peculiar species of dysentery."

Dr. Rogers, in his Essay on the Epidemic Diseases of Cork (1733), furnishes satisfactory evidence of the intimate connexion that subsisted between the endemic typhus of that city and a *malignant* form of Dysentery that prevailed there for several years. He remarks that the two diseases kept pace with each other, that they seemed to partake of the same common cause, and were best treated by much the same line of treatment; *viz.* the use of the most generous, warm, and active cordials; all antiplogistic and lowering remedies were found to be utterly inadmissible.

The testimony of Sir John Pringle, also, is very strong upon the question under consideration. After stating that dysentery always becomes much aggravated towards Autumn and is then apt to "become epidemic and infectious" (from which expression it may be fairly inferred that, previously, it was sporadic and non-infectious), he gives the following instance of the pernicious effects of crowding dysenteric patients together:

"The village of Feckenheim was taken for an hospital, into which about 1500 sick were sent from the camp, and of that number the greatest part ill of dysentery, by which means the air became so vitiated, that not only the rest of the patients, but the apothecaries, nurses, and other attendants, with most of the inhabitants of the village, were also infected. To this acceded a still more formidable disease, the hospital or gaol fever, an inseparable attendant of foul air from crowds and animal corruption. These two combined caused a great mortality; while on the other hand such as were seized with the dysentery *and not removed from the camp*, though wanting many conveniences, kept free from this malignant fever, and commonly did well." "At Feckenheim few escaped; for how mild or bad soever the flux was, this fever (the malignant) almost surely supervened: the petechial spots, blotches, parotids, frequent mortifications, contagiousness, and the great mortality, set forth its pestilential nature." P. 86.

Other passages might be quoted from Pringle's work in confirmation of the views here stated. For example, in one place he says:

"The most fatal sort of fever, which so often attends the dysentery of the army, though not *essential* to it, is the *hospital* or *gaol distemper*, which at all times infects foul and crowded wards, but never so much as when they contain men labouring under a putrid disease." In another place he takes notice of Degner's epidemic, and remarks: 'As to the violence of the symptoms mentioned by that author, I own it exceeds anything I have observed upon the first seizure, but when a number of men, *even with the most favourable cases*, have been crowded into the hospitals of the army, the dysentery has *then* appeared with all the virulence that it did at Nimeguen. Pringle further adds, that 'when mortification takes place, the distemper is most contagious, whether in producing a simple dysentery, or one combined with *the common hospital fever;*' and, as he says elsewhere, 'It is to be apprehended that when a single person is taken ill of any putrid disease (such as dysentery), &c., and lies in a small and close apartment, he may fall into this malignant fever.'" P. 87.

Equally striking is the evidence furnished by Grimm, in the account which he has given of three "epidemic and eventually infectious" (epidemici et *tandem* contagiosi) diseases which prevailed for upwards of three years, about the middle of last century, in one district of the Duchy of Saxe Weimar. The *first* of these diseases was a malignant Fever, which began in December 1758, and raged throughout the Spring of 1759; it then abated somewhat of its violence; but still, during the remainder of the year, all the prevailing diseases in the place exhibited a marked tendency to assuming a type of malignancy. The fever revived in the Winter and Spring months of 1760; in the August following, it was succeeded by another and more destructive pestilence, the Dysentery. This continued its ravages to the end of November, when it again passed into the malignant fever; and so striking was the connection between the two, that, as Grimm has observed, "the same fever might well be called exanthematic at one time, and dysenteric at another, according to the season of the year in which it prevailed." "I have seen," adds he, "petechial and miliary eruptions appear on dysenteric patients; while, on the other hand, the cases of fever were often accompanied with a most offensive diarrhœa like a dysentery." It only required a change in the weather or season to cause a transmutation of the one disease into the other. In one passage, Grimm speaks of the dysentery being "associated with its own malignant fever;" and, in another, he seems to regard the former as merely a symptom of the latter; for he says that "the primary disease is the fever, arising from a peculiar vitiation of the humours, and affecting the bowels more than the other viscera of the abdomen." No candid person, one should think, after reading Grimm's description of the epidemic which he saw, can hesitate for a moment in regarding it as a compound of Dysentery and malignant Typhus. If such, then, were the case, can we be surprised at its exhibiting a virulent infectiousness?—a property or element, which, to use the author's significant expression, was "both an effect and a cause of the disease."—This remark, it will be observed, is pregnant with much meaning.

The epidemic described by Roederer affords equally conclusive evidence on the point we are considering. In July of 1760, there was much intermittent fever (often of a malignant type) in the town (Goettingen) and its neighbourhood; this was followed in the next month by dysentery, which prevailed to about the beginning of Winter, when it gradually lapsed into the epidemic described by him under the appellation of *morbus mucosus;*—from the immense quantity of mucus that was found on dissection within the bowels, more especially the smaller ones. This acute mucous fever sometimes assumed a tertian type; but not unfrequently, more especially in the camp hospitals, it put on the characters of malignant bilious or putrid typhus. In March 1761, it became a true petechial fever, accompanied with either furious delirium or coma, and constituting what Roederer calls his "febris mucosa acuta maligna." He points out the intimate connection between this disease and the dysentery which immediately preceded it; indeed, he does not hesitate to regard both as springing from the intermittent fevers which had gone before; these producing the dysentery, and the dysentery producing the mucous disease. The two last diseases resembled each other not only in their symptoms, but also in respect of many of the cadaveric changes which they left behind; viz. inflammation of the villous coat of the bowels, gangrenous eschars on the inner surface of the large ones, livid spots on the liver, induration of the pancreas, and infarction (congestion) of the lungs being mentioned as appearances common to both. Besides the Fever and Dysentery that swept away so many of the wretched inhabitants of the town which was then occupied by a hostile garrison, itself invested by an enemy in the field, another, not uncommon, offspring of an atmosphere charged with morbific effluvia from a multitude of patients crowded together and suffering at the same time from insufficient food, viz. Hospital Gangrene, made its appearance among the wounded, and added to the horrors of the place.

Tissot, whom we have already quoted, says, in one passage: "If the corruption of humours, which creates malignant fevers, be united with the causes which produce dysentery, the dysentery resulting therefrom will be malignant;" and Geach, in his observations on the epidemic dysentery at Plymouth in 1781, tells us that, in some instances, "the patients died in a day or two after the seizure, and had *vibices* large and very black, followed with such a degree of putrefaction and stench as to deprive almost instantaneously two female attendants of their senses, who very soon after became also dysenteric, ran through the stages of the disorder, till at length one of them died, and the other with difficulty recovered."

On the subject of Typhoid Dysentery, Frank has well remarked;—"Whoever will place before his eyes a faithful description of typhus, and add thereto the phenomena of dysentery in the way of symptoms, will not require a more lengthened description of this dreadful disease (asthenic malignant dysentery)." He then enumerates some of the symptoms, such as "the sudden and extreme prostration of the vital powers, small pulse, tremor of the limbs, twitching of the tendons, spasms, drowsiness, delirium, with, not unfrequently, petechiæ and livid spots upon the skin. This species of the disease generally prevails in camps, besieged cities, on board ships, &c. It often produces terrible ravages, and is unquestionably infectious."

The details furnished by Cheyne of the dysentery which prevailed in Ireland, concurrently with the epidemic fever of 1817 and 1818, are highly instructive on many points connected with this combination of the disease. Dr. C. considered that both diseases originated from the same morbific causes.

Before concluding this part of his subject, Dr. Harty alludes to the remarkable decrease in the mortality in London from Dysentery (under which name used to be included not only the "bloody flux," but also the "griping of the guts," which we read of in the writings of the old physicians) that has taken place during the last two centuries. We gather from Dr. Heberden that, in the seventeenth century, the annual number of deaths from this disease alone in the Metropolis appear never to have been less than 1000, and in some years to have exceeded 4000 ; whereas, in the middle of the eighteenth, it was little more than 100, and at its close not more than 20. The following passage is replete with so much interest that we are glad of the opportunity of bringing it under the reader's notice.

"These facts relative to the gradual decline of dysentery, or rather of its mortality, in London, are not to be paralleled in the history of any other disease; the plague itself furnishes no parallel case, because that disease either raged violently or disappeared altogether: not so the dysentery, which has declined not so much, perhaps, in the numbers attacked, as in its positive mortality. The cause of so great an alteration in the health of the people of England, Heberden attributes to the improvements that have gradually taken place in London and all the great towns, and in the manner of living throughout the whole kingdom, particularly with respect to cleanliness and ventilation. The great influence of the assigned causes I by no means dispute; on the contrary, I am perfectly satisfied of their efficacy in the production of these happy results: in admitting this, however, I consider them to have been not the *immediate* but merely the *remote* agents in effecting such a change in the mortality of dysentery. The influence of these causes was exerted through the medium of another disease, which, if the preceding views be correct, is the great source of *danger* as well as *contagion* in dysentery: their operation was primarily exerted in mitigating and preventing contagious fever; by that means acting secondarily on dysentery, which, when separated from this its dangerous associate, is no longer an object of terror to the patient or his attendants. This opinion is supported by various facts relative to the plague, to be found in the same author. It would appear that the disease, so called, was seldom absent from London previously to the great fire of 1666, after which event it never more visited the metropolis ; it appeared also that dysentery, antecedently to that period, was in general both malignant and destructive, and that shortly after there was a wonderful decrease in its frequency and severity; and though dysentery still prevailed from that time to 1692, a space of twenty-five years, it had much decreased in mortality. Morton states it to have been epidemic from 1666 to 1672, and at first exceedingly fatal (300 or 400 dying every week during its *acme)*, though less so towards the end of that period ; from Sydenham's description, dysentery appears, except occasionally, to have been devoid of contagion; and Willis's History of the Dysenteria Cruenta of the year 1670 coincides generally with this statement.

"Now I consider it almost demonstrated by Heberden, that the plague of London was nothing more than the malignant contagious fever, exalted by various auxiliary aids to such a pitch of destructive violence as well to merit that name; and that the identity of the two diseases is satisfactorily supported by authority and facts. The singularly rapid decline in the mortality of dysentery, when the plague ceased to visit London, or rather when fevers lost much of their malignity, cannot but lead us to conclude that the immediate cause of this change was owing to the diminished influence and dominion of malignant fever in London. We may indeed take it as an admitted fact, that the malignity of dysentery has ceased with the disappearance of the plague. What so common among medical writers, down to the eighteenth century, as accounts of malignant and contagious dysentery? Do not their writings abound with concurrent histories of the plague or of very malignant fevers? Diemerbroeck, for example, mentions, amongst the precursors of the plague which ravaged Nimeguen in 1636: 'Morbi epidemii mali moris, ut erant variolæ, morbilli, *dysenteriæ* valde

*malignæ* et contagiosæ, imprimis febres putridæ, malignissimæ et purpuratæ, plurimisque lethales.' "* P. 101.

Having now demonstrated, beyond all possibility of dispute, the existence of a typhoid form or species of Dysentery, it only remains to be stated that this, the worst and most destructive, variety of the disease is not, like the remittent or intermittent form, limited to the Summer and Autumnal months, but may continue its ravages throughout the Winter; "being dependent, not so much on the seasons as on the febrile contagion, and continuing so long as the causes of the fever continue to operate." "This fever," continues Dr. Harty, "may be either originally a malignant typhus, or it may have been a remittent of bad character, converted by the peculiar circumstances of the sick into one of a contagious continued type." The reader will excuse us if we call his attention to the position here laid down; viz. that the accompanying fever of epidemic dysentery may be of a periodic type at first, and subsequently—from the operation of various insalubrious circumstances—assume that of genuine typhus. By bearing this fact in mind, he will be able to reconcile much of the conflicting, and seemingly contradictory evidence that has been adduced on the question of the infectiousness of particular epidemics.

Surely then, from all the facts and testimonies that have been adduced, there is good reason to believe that the division of Dysentery into three species, according as the intestinal affection is unattended with idiopathic fever, or as the accompanying fever is of a periodic or of a continued type, is one that is amply borne out by an impartial examination of the history of the disease, as given by the most experienced writers upon the subject. Hoffman, more than a century ago, had pointed out this three-fold division, as is evident from the following statement at the close of his description of an epidemic dysentery in 1726. "Three classes or grades of the disease might be observed. The *first* was very mild, attended with only flying rigors and heats, and did not always necessitate the patient to keep his bed; the *second*, somewhat malignant, was accompanied with fever, this being either of a quartan or of a semi-tertian type; and the *third*, of a genuinely malignant character, was associated with an acute, and, as it were, inflammatory fever; the symptoms being of the most violent description: this form was highly infectious, and proved rapidly fatal in a very large majority of cases." Of this last-mentioned form he afterwards

---

* It would seem, from the following passage in Diemerbroeck's treatise *de Peste*, that he took nearly the same view, as to the nature of this disease, with that which our author has expressed in the passage now quoted. From this account it appears that, "after an intensely hot dry Summer, a pestilential fever broke out at Nimeguen and other places in Gueldres, causing great mortality. In Autumn, the heat and draught still being excessive, various febrile diseases, such as small-pox, measles, and dysentery, all of a malignant and putrid character, made their appearance in different parts; but chief and foremost was the pestilential fever which, daily becoming more widely diffused, gradually assumed a more aggravated character, until at length *in apertissimam pestem transiret.*" The intimate connection (says Dr. Harty) of the three diseases—Fever, Dysentery, and Plague—almost simultaneously raging, is here well and clearly marked.

says that the accompanying fever, " more dangerous than the dysantery itself, was of a malignant petechial description."

The testimony of Sir James Macgregor confirms the strict accuracy of Hoffman's division; for, in the account which he has given of the dysentery which prevailed among the British troops during the Peninsular campaigns, he distinctly asserts that, in some cases the disease, from its commencement, appeared to be unattended with fever, and, in a short time, ran into the chronic stage. In very many cases, the disease was connected with intermittent fevers, and these indeed were frequently observed to terminate in dysentery. " The type of fever, however," he continues, " accompanying dysentery was very much modified by that of the prevailing epidemic. In the hospitals in the Alentijo and Estremadura in 1812, intermittent fever prevailed or accompanied dysentery, and remittent fever, when the army advanced so rapidly and remained some time stationary in the two Castiles, in July, August, and September. Every case of dysentery which appeared in the battalions of the Guards in 1812 and 1813 was accompanied by the *typhus gravior*, and very generally had a fatal termination, as did many at Ciudad Rodrigo, &c., where the same form of fever was prevalent." At that place, the situation of which is unhealthy, aggravated by the interment of 20,000 bodies in the course of a few months, the mortality was great.

Granting now that the position with which we started—viz. the threefold division of Dysentery, according as it is unattended with idiopathic fever, or is associated with periodic or with continued fever—has been satisfactorily made out, it will not be necessary for us to occupy any space with proving that it is the last-mentioned form or species of the disease which is liable to exhibit infectious properties. A few short extracts from two or three of the leading authorities will be all that we can afford room for. Zimmermann has, in one place, remarked that " it appeared that our dysentery in general became contagious purely through nastiness and the crowding many people together in a small space, but was by no means *so itself;* for, though many were attacked with it at once, this seems to proceed from a more universal and widely-different cause, which operated at once upon every one."

In another passage he observes:

" ' The benignant species of dysentery becomes contagious, malignant, and extremely dangerous, when many sick people are crowded together in a small space, or when peculiar external or internal causes produce malignity in particular persons:' and he adds, that he does not see ' that camp dysenteries are in themselves more malignant than those that happen in cities, although in the army and military hospitals they become excessively malignant and contagious from several circumstances: the same, however, takes place in cities, when a great quantity of people, attacked with this disease, are crowded together in a small space, or where the other different causes subsist of a peculiar or general malignity.' And in p. 151 he says: ' *When hospitals are filled with dysenteric people, some of the assistants are attacked only with dysentery, and others with the gaol or hospital fever, that ends in bloody and gangrenous stools.*' ' From all these observations, made partly by me, and partly by other physicians, I conclude,' says Zimmermann, ' that the dysentery is very often only *accidentally* contagious,' but that it also frequently becomes essentially so just before the death of the patient." P. 134.

Hillary, after describing the endemic dysentery of Barbadoes, and attributing it to the atmospheric vicissitudes of that climate in particular months of the year, goes on to say:

"It is also probable that it may be sometimes produced by *infectious miasmata,* exhaled from diseased bodies, and floating in the air, which are received into the mouth when we breathe, stick there to the saliva, and are carried with it down into the stomach and intestines, where they produce all the above-mentioned symptoms, when they meet with a constitution fitted by the above-mentioned causes to receive those infectious effluvia, and to produce the disease." "And thus the disease becomes both epidemical and contagious, though it was not the latter at its first invasion, or seizing the first patient. This I have often observed, especially when *great numbers* have laboured under it at the *same time,* as often happens among the *negroes.*" P. 116.

Diemerbroeck, who unequivocally maintains that the dysentery, which prevailed in Nimeguen at the same time with the pestilential fever, of which he has given so accurate a description, was malignant and infectious, subsequently mentions another epidemic of the same disease, whose spread was (he says) owing not to infection, but to an unhealthy character of the season, in conjunction with the bad description of food that the lower classes lived upon. Frank very distinctly attributes the infectiousness (when present) of dysentery to the nature of the fever with which it happens to be associated: in other cases, the disease is, in his opinion, not communicable from one person to another. The late Dr. Fergusson,* whose experience of the disease in different climates was very extensive, says:—"Dysentery, I believe, I can declare to be in no case contagious. I never saw anything like it except in Holland, after the weather became cold, when it was seen as a local symptom, or irregular form of typhus fever: but more could not then be said than that it was about to be swallowed up in the prevailing epidemic, and *formed a combination* rather than a distinct type of disease." The same sentiment is reiterated in the article on Dysentery in his recently-published posthumous volume, entitled "Notes and Recollections of a Professional Life." Another of our late army physicians, Dr. Somers, has expressed an entirely similar opinion. He denies a specific contagion to the disease, and states that he "never witnessed the production of contagion by *pure unmixed* dysentery." He had, however, met with some cases "in which low fever and chronic dysentery were co-existent: in those, unquestionably, there was contagious disease; but, this evidently arose from the superinduction of typhus upon dysentery."

These passages will doubtless suffice to convince every candid reader that Dysentery is liable, under certain circumstances which have been specified, to become infectious, or transmissible from one individual to another. It will be seen from the following extract that, the views maintained by Dr. Copland on this important subject, are not exactly those for which our author contends:

"Dr. C. endeavours indirectly to negative the propositions I have been contending for, that the combination of dysentery with typhus is the special cause productive of contagion in the former disease:

---

* *Medico-Chirurgical Transactions,* Vol. 2.

'Many writers,' he says, 'conceive that the asthenic varieties are complications of simple dysentery with different kinds of fever, and that when they are infectious it is not the dysentery but the fever which possesses this property. *Some* authors suppose that the typhoid variety especially is a complication of this description. But if such,' he adds, 'be the case, wherefore should the disorder which is communicated be always dysentery and not fever? Moreover, this form of dysentery is often present where a case of typhus cannot be found.' He then summarily disposes of the whole question by asserting 'the fact as incontrovertible, that the asthenic forms are direct and necessary, and uniform results of certain diversified but concurrent causes, and not contingent associations of two diseases capable of separate existences.' The fact thus laid down as 'incontrovertible' being not merely a *petitio principi*, but the very assertion of the questions at issue, I shall pass it by, and proceed to examine his two arguments; and *first* as to the inference he would have drawn from the questions above quoted: 'Wherefore should the disorder which is communicated be *always dysentery* and *not* fever?' The question is doubly erroneous, inasmuch as the disease sometimes communicated is the fever and not the dysentery; as, for example, when, according to Zimmermann, hospitals are filled with dysenteric people, some of the assistants were attacked only with the dysentery, and others with the gaol or hospital fever; and that 'this fever may attack people in health without being attended with the dysentery, though it arise from the putrid and confined vapours of that distemper.'* Again, the dysentery that is communicated is not dysentery merely, but dysentery in combination with the fever. *This* assertion of an 'incontrovertible fact' is amply borne out by the multifarious authorities already quoted, such as Pringle, Hoffman, Zimmermann, Vignes, Grimm, Frank, &c. His *second* argument consists in the statement that 'this [the typhoid] form of dysentery is often present where a case of typhus cannot be found.' Now for this statement no authority is given, such as might be investigated; but is not Dr. C. well aware that, under favourable circumstances (such as those recapitulated by Frank and others), there is no disease more readily superinduced or spontaneously generated than typhus, a position fully supported by the several authorities already quoted in illustration of this very typhoid variety, and which I have myself further illustrated in my work on the Epidemic Contagious Fever of Ireland (see p. 161, *et seq.*) All these authorities distinctly shew that the circumstances under which contagious dysentery rages, are the same with those which give origin or aggravation to the prevalence of typhus." P. 140.

We have left ourselves no room for any observations on the Treatment of Dysentery, although the consideration of this part of the subject is necessarily fraught with most interest. But as no sound therapeutic principles can be laid down in reference to any disease, unless we have clear notions as to the real nature of the enemy that we have to contend with, the preceding pages will not be without some interest to the practical physician, if they serve in any degree to teach him how to discriminate, with greater accuracy than before, the kind of dysentery which he may be called upon hereafter to treat. At all events, they will enable him to account for many of the conflicting statements to be found in the

---

* "Nous ferons remarquer," says Vignes, "que l'infection d'une maladie ne produit pas toujours la meme espece d'affection: l'infection de la dysenterie peut, par exemple, occasionner une indisposition bilieuse ou une fievre adynamique, &c.; de meme qu'on peut etre affecté de celle-ci (la dysenterie) par toute autre exhalaison deletere que celle des dysenteriques."

writings of different authors, upon some of the most important points in the general history of the disease. They will show him the reason why a rigid antiphlogistic treatment may be required at one time or in one set of cases; why at another time, and in another locality, no cure can be effected without the aid of bark; and why, in a third set of cases, the chief reliance must be placed upon the adoption of such hygienic measures as will secure cleanliness, pure air, a regular supply of light, nourishing food, and, it may be, the cotemporaneous use of wine and other cordials. For particulars upon these matters, we must refer the reader to Dr. Harty's work, which, we hesitate not to say, should be carefully perused by every member of the profession without exception, from the experienced veteran to him who is just entering upon the duties of active practice. To the young physician it furnishes an admirable pattern of what a medical book ought to be; not the hasty offspring of an impatient ambition, eager for notoriety, it matters little how obtained; but the well-considered production of a studious and philosophic mind, whose chief aim has been to benefit medicine, and promote the welfare of mankind.

